# Catchment scale runoff time-series generation and validation using statistical models for the Continental United States

**DOI:** 10.1016/j.envsoft.2022.105321

**Published:** 2022-03-01

**Authors:** Douglas Patton, Deron Smith, Muluken E. Muche, Kurt Wolfe, Rajbir Parmar, John M. Johnston

**Affiliations:** a Oak Ridge Institute of Science and Education, USA; b Environmental Protection Agency, USA; c Oak Ridge Institute of Science and Education, National Science Foundation, USA

## Abstract

We developed statistical models to generate runoff time-series at National Hydrography Dataset Plus Version 2 (NHDPlusV2) catchment scale for the Continental United States (CONUS). The models use Normalized Difference Vegetation Index (NDVI) based Curve Number (CN) to generate initial runoff time-series which then is corrected using statistical models to improve accuracy. We used the North American Land Data Assimilation System 2 (NLDAS-2) catchment scale runoff time-series as the reference data for model training and validation. We used 17 years of 16-day, 250-m resolution NDVI data as a proxy for hydrologic conditions during a representative year to calculate 23 NDVI based-CN (NDVI-CN) values for each of 2.65 million NHDPlusV2 catchments for the Contiguous U.S. To maximize predictive accuracy while avoiding optimistically biased model validation results, we developed a spatio-temporal cross-validation framework for estimating, selecting, and validating the statistical correction models. We found that in many of the physiographic sections comprising CONUS, even simple linear regression models were highly effective at correcting NDVI-CN runoff to achieve Nash-Sutcliffe Efficiency values above 0.5. However, all models showed poor performance in physiographic sections that experience significant snow accumulation.

## 1. Introduction

Effective management of hydrologic resources and hazards often depends on accurate simulations of runoff. For example, runoff time series can be combined with other environmental data to characterize how a system responds to various climate and land use scenarios. To facilitate the work of researchers and managers seeking to understand and manage hydrologic systems, we developed an automated Curve Number (CN) based technique for estimating catchment level runoff that allows for the use of either simulated or historical data for precipitation and landcover. To facilitate validation of the models developed in this paper for various applications, we designed and implemented a machine learning accuracy assessment framework that withholds validation data from statistical model training both spatially and temporally to build confidence that the resulting accuracy measures truly characterize how the models generalize to other catchments and time periods.

To implement the accuracy assessment, this paper presents a machine-learning framework for a state-of-the-art, approximately unbiased approach to quantifying predictive accuracy in a hydrologic spatio-temporal context. Using an existing automated technique for quantifying hydrologic condition (Muche et al., 2019a; 2019b), we generated NHDPlusV2 catchment-scale CNs for CONUS and applied the framework to estimate and evaluate a variety of relatively simple CN-generated runoff time-series correction models that often dramatically improve runoff accuracy. Additionally, because the technique we employed for automating CN generation adheres to the conventional CN approach, we expect that the correction models are likely to enhance the accuracy of runoff time series generated by any of the variants of CN, such as the recent GCN250 (Jaafar et al., 2019).

### 1.1. Hydrologic runoff modeling

A variety of research topics involve data modeling that requires information about how precipitation patterns translate into measures such as runoff and streamflow. Historical runoff estimates utilizing a robust set of environmental forcing variables have been made readily available through the North American Land Data Assimilation System (NLDAS) (Xia et al., 2013) and the Global Land Data Assimilation System GLDAS (Rodell et al., 2004) Land Surface Model (LSM) projects, for example. Historical data are useful for assessing and training models, but these projects do not provide a means for simulating runoff in counterfactual or future conditions.

A variety of approaches have been developed to estimate the relationship between hydrologically relevant environmental variables such as precipitation and runoff. Sitterson et al. (2017) provide a taxonomy of rainfall-runoff models based primarily on the correspondence of the model with physical reality and spatial resolution. At one end of the spectrum, the CN methods of runoff modeling offer analysts one of the simplest approaches to runoff modeling. At the other end of the spectrum are multi-input, multi-output LSMs such as NLDAS-2 (henceforth referred to as NLDAS), which itself is an ensemble of LSMs (Xia et al., 2012a; 2012b).

Other runoff modeling approaches include the Geomorphological Instantaneous Unit Hydrograph approach, a more recent and a more technically sophisticated approach to rainfall-runoff modeling (Rigon et al., 2016). Recently, Oppel and Schumann (2020) applied machine learning estimators to explore transferability of geomorphological instant unit hydrograph runoff models between catchments based on catchment characteristics and a basin classification scheme derived from their models. Fractal geometry has been applied to modeling surface runoff (Gires et al., 2018). Another group of runoff models is the “GR chain” (Ficchì et al., 2019) that vary in temporal resolution, which Ficchì et al., (2019) extended to include flux-matching criteria.

The Soil Conservation Service-Curve Number also widely referred to as the Curve Number method was developed by the United States Department of Agriculture (USDA) in the 1950s to predict direct runoff from rainfall events and it is a widely adopted method in surface runoff estimation (Hawkins et al., 2008; Hawkins 2014; Lian et al., 2020). The method was developed using measured rainfall and runoff data from several agricultural research watersheds primarily in the Eastern, Midwestern, and Southern U.S.; the rainfall-runoff relationship in the study watersheds was extrapolated to an empirical number (the Curve Number) using land use/cover, hydrologic soil groups, and hydrologic conditions of watersheds (Hawkins et al., 2008, Muche et al., 2019a; Rallison 1980). The CN method has been globally adapted to areas with varying land use/cover, soil properties, and climatic conditions. It has also been incorporated into various continuous hydrologic/watershed models though the method was originally devised for event-based rainfall-runoff modeling (Garen and Moore 2005; Hawkins 1996; Kennan et al. 2007; Muche et al., 2019a). Despite wide applications of the CN method, some watersheds have been found to exhibit significant differences between observed and predicted runoff using the CN method (Hawkins 2014; Muche et al., 2019a). The effects of rainfall volume, intensity, and frequency (Muche et al., 2019a; Wang and Bi 2020), in addition to the seasonality of rainfall-runoff relationship could be among the contributing factors to the CN method’s low accuracy in those watersheds (Rodríguez-Blanco et al., 2012). To increase the accuracy of CN generated runoff, Silveira et al. (2000) incorporated automated estimation of antecedent moisture using five days of lagged rainfall. Recently, advancements in Geographic Information Science (GIS) created the opportunity to account for seasonality in the rainfall-runoff relationship by using remote sensed data to flexibly approximate hydrologic condition (Muche 2016; Nasiri and Alipur 2014; Singh and Khare 2021). Moderate Resolution Imaging Spectroradiometer-Normalized Difference Vegetation Index (MODIS-NDVI) were applied to CN estimation by several authors (Gandini and Usunoff 2004; Muche et al., 2019a, 2019b; Nasiri and Alipur 2014; Singh and Khare 2021). Muche et al. (2019a) used MODIS-NDVI to estimate CN using 12 years of observed rainfall and runoff at four small watersheds in the Konza Prairie Long-term Ecological Research site. Muche et al. (2019b) extended this work, using MODIS-NDVI for catchment-level CN development spanning CONUS as part of USEPA’s Hydrologic Micro Services (HMS) computational platform, which is used in the results below.

### 1.2. Model validation and selection

Validation is generally regarded as an important step in modeling, though it is not clear what exactly is meant by validation and what one must do to achieve it. Schlesinger (1979) defined validation for computerized simulations only in terms of comparison to reality in the *domain of applicability*. Schruben (1980) discussed simulation *credibility* as a more practical standard to meet than *strict validation*, requiring simulation output to be indistinguishable from observations of reality by a human in the same manner as a Turing Test for artificial human intelligence. Sargent (2013) discusses validation broadly for empirical studies and describes several *Validation Techniques* including *inner validation* as an assessment process using data resampling and *historical data validation* as a process of splitting data into *building* and *testing* sets.

Klemeš (1986) offers an early and widely cited guide to validation of hydrologic models that generally corresponds well with modern machine learning approaches to model validation. Biondi et al. (2012) review and refine validation concepts in hydrology, and they discuss *performance* or *model validation*, which includes qualitative assessment of graphs and quantitative assessments of model metrics on split-samples. They also discuss a distinct type of validation, *scientific validation*, wherein one considers the theoretical underpinnings of the model. Common machine learning terminology contrasts with the above uses of *validation*. Hastie et al. (2017) uses the *validation set* for model selection and a final *testing set* for quantifying generalization error, which is called *validation* in the above contexts other than machine learning. In machine learning, a great deal of attention is paid to using validation-like metrics for both model selection and validation, typically with distinct treatments of data.

Common statistical approaches to model selection that rely on error estimates from training observations can lead to substantial downward bias in error metrics, leading to overly optimistic conclusions about model accuracy (Picard and Cook 1984). Cross-validation approaches to model validation split a dataset into *n* folds, use *n* − 1 of the folds to train a model, quantify predictive accuracy on the withheld fold, and cycle through all *n* folds, generating an empirical distribution of model accuracy that approximates expected prediction error (Hastie et al., 2017, p254). Hawkins et al. (2003) favor cross-validation approaches to model selection and validation rather than a single hold-out test set because cross-validation balances the desire for more data with the need for quantifying predictive accuracy. However, cross-validation when done incorrectly can still lead to biased performance measures and inferior model selection (Cawley and Talbot 2010; Hastie et al., 2017 p245).

Guyon et al. (2010) provide a useful discussion of the nested relationship between the two (or more) types of parameters in the context of diverse statistical learners. In this process of “multi-level inference” that Guyon et al. (2010) describe, parameters are chosen in an inner estimation step by analytically efficient learning algorithms (e.g., linear algebra solutions for Ordinary Least Squares (OLS) linear regression) and hyper-parameters are chosen by repeatedly invoking the efficient algorithms with different hyper-parameter values. For multi-level inference approaches, cross-validation serves as the framework for each level of inference to avoid over-fitting (Cawley and Talbot 2010).

Fushiki (2011) found for regression problems that n-fold cross validation may bias prediction error upwards while training error is a downward biased estimate of prediction error. Hastie et al. (2017, p254) characterize 10-fold cross-validation as an “approximately unbiased” means of quantifying expected or extra-sample error. Techniques such as 5-fold and 10-fold cross-validation are computationally efficient approaches for quantifying prediction error with generally lower variance than leave-one-out cross-validation (Hastie et al., 2017 p255). In classification problems, n-fold cross validation has exhibited reduced bias and computational complexity relative to the bootstrap (Kim 2009), an alternative to cross-validation.

Time series datasets require additional assumptions to justify cross-validation. More conservative approaches to validation of time series models typically require withholding later observations in the dataset from training for testing the model’s performance with new data. This process has been called “last block validation” (Bergmeir and Benítez 2012) or “out-of-sample evaluation” (Bergmeir et al., 2018). Recent advances have opened up possibilities for efficient cross-validation with some time series estimators, which is particularly adventitious for small datasets (Bergmeir et al., 2018) because all data can be used for validation.

Data can be grouped for modeling and assessment in a variety of ways. In machine learning, the term, *slice* (Chung et al., 2019), refers to divisions in the data by predictor variables that can be used to assess model performance with greater granularity. Validation metrics that group slices together can potentially obscure poorly performing slices (Chung et al., 2019). The idea of assessing slice performance is closely related to the idea of *transportability* in hydrology as discussed by Klemeš (1986), who described an early cross-validation-like testing procedure for detecting poor performing members of a group of catchments to validate simulations in ungauged members of the group.

The model scoring or loss metric also plays an important role in model selection and validation. Gupta et al. (2009) applied and refined decompositions of the popular Nash-Sutcliffe Efficiency (NSE) model scoring metric in the context of runoff simulations, criticizing models optimized with NSE as the score for being of use only in *normal* conditions. This problem can be remediated to some extent by their proposed Kling-Gupta Efficiency (KGE) metrics (Gupta et al., 2009). Knoben et al. (2019) point out interpretation issues associated with several parametric and non-parametric variants of KGE; they emphasize a lack of a clear benchmark or cutoff value with KGE metrics, while the NSE value of zero benchmarks simulation performance against the mean of the observed series.

### 1.3. Corrective modeling

Models that correct simulations have been developed extensively in the earth sciences. Watson (2019) discusses the tradeoffs associated with physical versus purely data-driven approaches to increasing predictive accuracy at short and long timescales in the context of climate simulations. Dinge et al. (2019) distinguish between *point to point* correction models and models that use time series characteristics to increase performance in the context of applying error correction models to wind speed prediction. Zjavka (2015) applies a polynomial neural network to correct wind speed using lagged measures of nearby environmental variables.

### 1.4. Regression prediction

We use a broad definition of regression from Hastie et al. (2017, p10), which encompasses any statistical learner that makes quantitative predictions. In practice, regression models have a continuous dependent variable in contrast to classification and ordered categorical models with discrete dependent variables. Accordingly, the linear regressions, regularized linear regressions, and gradient boosting ensembles are all referred to as regressors or regression models.

Regularized linear regression estimators share similarities with OLS, but with additional structure to reduce the variability (increase the stability) of the parameter estimates, at the expense of increased bias (Frank and Friedman 1993). The Lasso regularized regression effectively selects predictor variables, pushing some coefficient estimates to zero, while the Ridge regularized regression tends to push regression coefficients towards equality with each other (Tibshirani 1996). The Group Lasso was developed to select groups of dummy variables for multi-category predictors, and extensions to the Group Lasso have been developed to preserve hierarchal connection between interaction and main effects in lasso regression models (Lim and Hastie, 2015). In contrast to OLS, the basic Lasso estimator can more generally estimate flexible dummy variable specifications with overlapping categories as discussed in Lim and Hastie (2015). The elastic-net regressor combines the strengths of the Lasso and Ridge regression estimators, with the ability to model high collinearity among variables like Ridge and the ability to do variable selection like Lasso (Zou and Hastie, 2005).

The gradient boosting regressor is an extension of the gradient boosting classifier and has been described in detail by Friedman (2001) and by Hastie et al. (2017). The algorithm fits an additive sequence of simple regression tree estimators with the gradient of the previous estimator’s loss function used as the dependent variable for training the subsequent tree in an iterative procedure. According to Guyon et al. (2010), boosting methods of regression are less vulnerable to overfitting because they minimize a guaranteed risk function. Hastie et al. (2017, p340) quote others in describing the classifier version of gradient boosting as the “best off-the-shelf classifier in the world”.

## 2. Methods

### 2.1. NDVI-based automated curve number development

The primary challenge in automating the generation of runoff time series using the Curve Number method is the selection of the hydrologic condition. The hydrologic condition functions as a categorical variable taking into consideration several possible influencing factors mainly related to land-cover type at the time of precipitation event. The customary approach to specifying hydrologic condition requires site specific expert analysis that hinders scaling the approach to larger areas. Remote sensing data has been shown to be a viable approach to specifying hydrologic condition, facilitating automated estimation of CN values. We followed the work of Muche et al. (2019a, 2019b) and used 250-m, 16 day resolution MODIS NDVI (Didan 2015) data along with land cover and soil data to quantify hydrologic condition and create a time series of twenty-three CN values spanning an average year for each of approximately 2.65 million NHDPlusV2 catchments in CONUS.

To compute these numerous CN values, we first needed to quantify the corresponding hydrologic condition. We used Google Earth Engine to obtain and spatially average seventeen years (2001–2017) of MODIS NDVI satellite raster data to the NHDPlusV2 catchment. Next for each catchment and each of twenty-three annual, sixteen-day timesteps, we temporally averaged the 17 observations of spatially averaged NDVI. We then used these time and space averaged NDVI values along with NLCD land-cover data (discussed next) for each catchment and time period to determine the hydrologic condition as Poor, Normal or Good based on the ranges specified in Table 1.

We obtained catchment level NLCD 2011 land cover data and STATSGO derived sand and clay soil composition percentages from the EPA StreamCat dataset (Hill et al., 2016). We used the STATSGO percentages to determine the hydrologic soil group of each catchment. Finally, for each timestep and each catchment we used the land cover, hydrologic soil group, and hydrologic condition values along with the USDA’s Soil Conservation Service curve number tables to obtain NDVI-CN values for each catchment and each of the 23 annual 16 day time periods. For each catchment and each timestep, the NDVI and CN values as well as annual average CN values can be obtained at ftp://newftp.epa.gov/exposure/CurveNumberNDVI. Additionally, the spatially and temporally averaged NDVI data can be obtained for each catchment at https://qed.epa.gov/hms/rest/api/info/catchment?cn=true&comid=COMID, where COMID is replaced by a NHDPlus catchment ID (e.g., https://qed.epa.gov/hms/rest/api/info/catchment?cn=true&comid=331416).

### 2.2. Accuracy assessment

We used the CN values described above to develop a runoff database to investigate the accuracy of NDVI-CN generated daily runoff using NLDAS runoff as the target. For the spatial units in our database we randomly selected 5 NHDPlus catchments in each United States Geologic Survey (USGS) physiographic section (Fenneman and Johnson 1946). Next, we retrieved 17 years of NLDAS runoff data and NDVI-CN runoff data (forced by NLDAS precipitation data) for each catchment. We also retrieved the GLDAS runoff and NDVI-CN runoff (forced by GLDAS precipitation data) and present parallel, condensed results based on that data in Appendix 1.

In the discussion of model selection and validation below, we follow the bulk of the empirical literature and reserve the term *validation* to describe the final split of data that is not used for any kind of model selection (in this paper), but only for reporting a final estimate of predictive accuracy. This decision contrasts with recent trends in machine learning research, where validation data are used for model selection and testing data are used for quantifying predictive accuracy of the selected model (e.g., Hastie et al., 2017). We use the term *validate* or *validation* to characterize the final chronological split of data as well as the subsequent accuracy analysis. This *validation* stage provides information not used for model selection in this paper but that is developed for use by end-users who require information about expected predictive accuracy. We use the term *test* or *testing* more generically to refer to any accuracy assessment such as those performed during model selection. Validation is thus a special kind of model testing deliberately designed to avoid data leakage that can optimistically bias results. We adopt this terminology for consistency with the hydrologic literature (and a number of other empirical sciences as well).

The purpose of assessing the accuracy of simulated runoff relative to the target runoff is to provide information to users about the likely quality of future simulations that may include times and locations not present in our database. To achieve this, we develop a validation approach with a resampling design based on holding-out observations for testing based on both time and space.

**Fig. 1.**
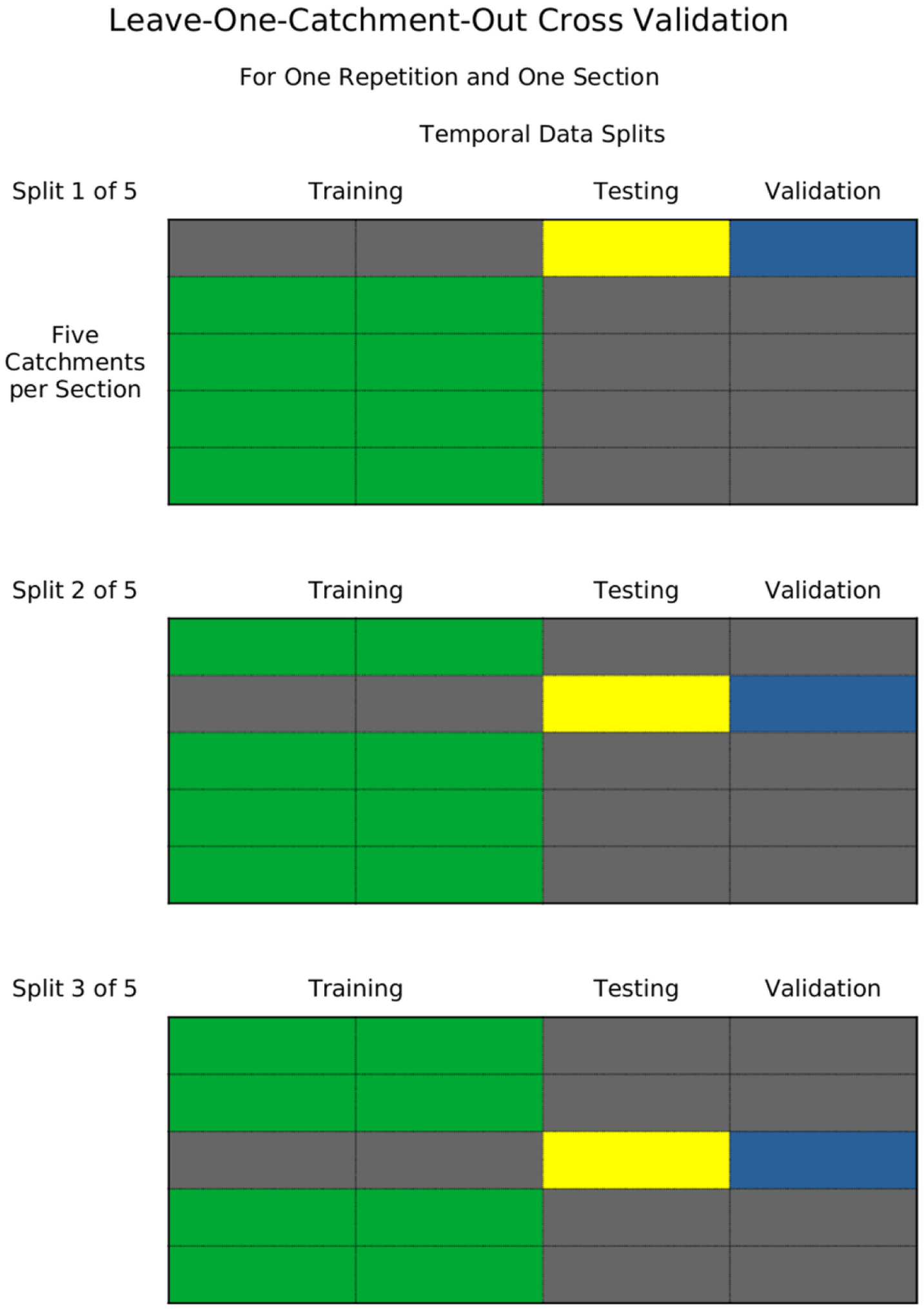
A diagram of the chronological splitting of data for model selection and validation.

First, because some of our runoff simulation models use lengthy time series of runoff data for training, we employ a traditional three-part temporal splitting of each time series. As can be seen in Fig. 1, the first half of a runoff time series is reserved for training the models, and the second half is split into a testing series used for final model selection and a validation series used for quantifying predictive accuracy. This approach helps ensure that the validation accuracy assessment of the models generalizes to other time periods, particularly the near future. This type of validation is an example of what Klemeš (1986) called a *split-sample test*.

**Fig. 2.**
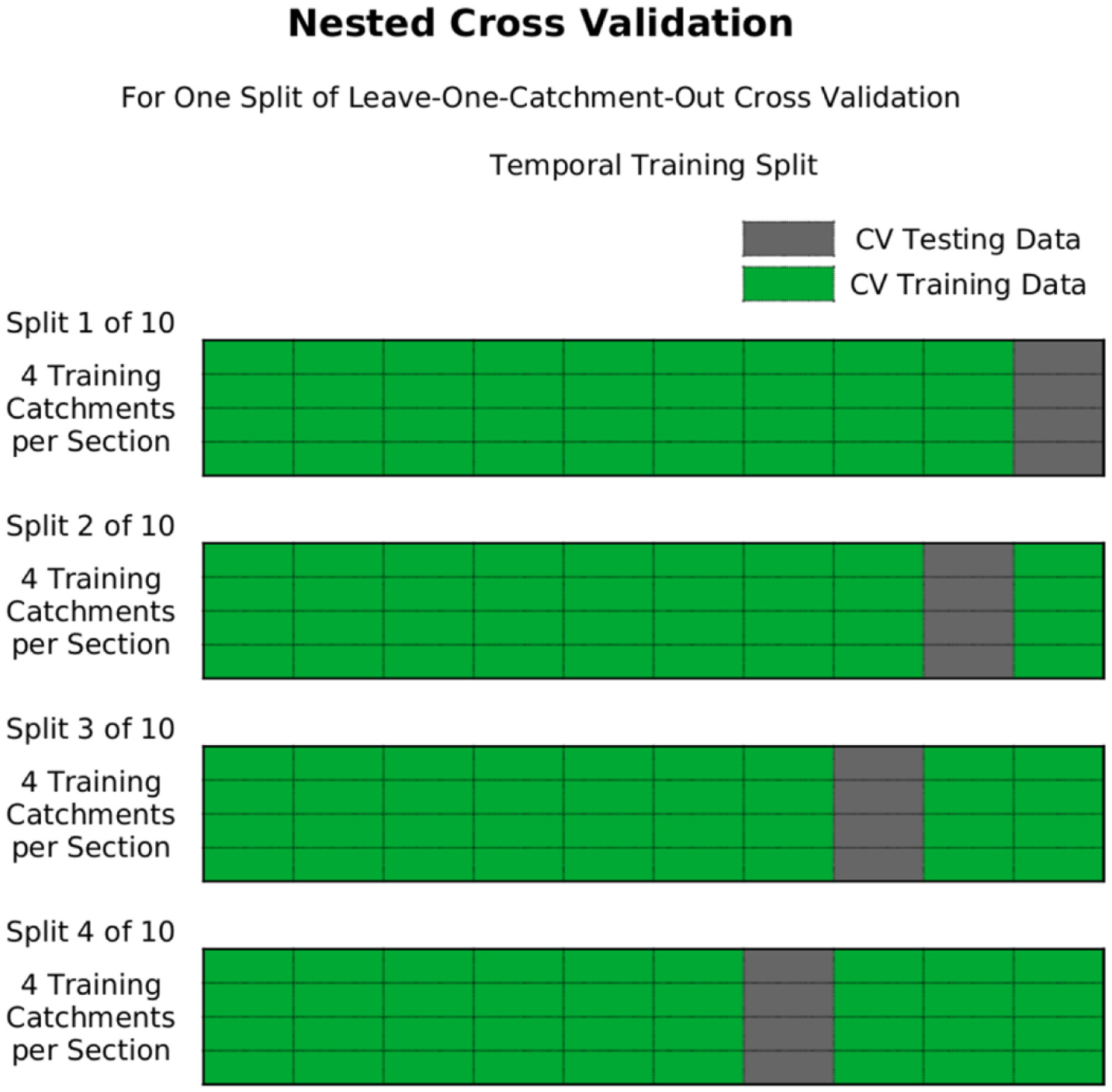
A diagram of the internal nested leave one catchment out cross-validation model assessment.

Second, for the runoff correction models that we develop below, there is also a potential concern about the ability of the accuracy metrics to generalize to catchments excluded from model training, such as those not in the runoff database. Accordingly, we evaluate predictive accuracy of each correction model using the average of a *leave-one-catchment-out-of-each-section* repeated cross validation approach. As shown in Fig. 2, the splitting algorithm we developed takes each physiographic section and places the five sampled catchments in a list. For a single repetition of cross-validation, each list is shuffled and then the first catchment in each list is excluded from the training data of a sub-model and reserved for testing that model. The next training/test split comes from excluding the second catchment in each list and so on until each of the 5 catchments in each section have been reserved from training a model and used for testing and validation of that model. This procedure is repeated to ensure the accuracy metric does not depend on any patterns in the test-data from a single shuffling of the catchments in a physiographic section. This approach to geographic data-splitting is in addition to the single chronological split discussed above and is an example of what Klemeš (1986) referred to as a *proxy-basin test*. The result is that no catchment or time period is ever present in both training and test or validation data for any of the prediction accuracy measures that we report.

The simulated runoff series, and the target or observed runoff series, y_obs_, we are comparing are continuous and non-negative, allowing for a wide range of accuracy measures based on differences or similarities between the two series. We select Pearson’s correlation coefficient (r_p_) and Nash-Sutcliffe Efficiency (NSE) because these two measures are popular in both machine learning and hydrology applications and because the two measures inform us about distinct aspects of model fit. Nash-Sutcliffe Efficiency is identical to the familiar coefficient of determination or R^2^ from a linear regression where y_obs_ is the dependent variable and y_sim_ is treated as the regression prediction. NSE can be applied to non-linear models with a potential range from minus infinity to positive one. Negative values for NSE indicate that the mean of the target is a better predictor of the target than the simulated series. The magnitude of r_p_ can range from zero to one, measuring how close a linear transformation of the simulation is to the target.

Comparing NSE to r_p_ is helpful for illustrating the contrasting properties of these two measures. NSE effectively benchmarks the simulation against the target’s mean, and importantly the mean of the target is not known at the time of the simulation. Further, the NSE does not center or rescale the simulation series to help it match the target series. In contrast, r_p_ benefits from a linear transformation of the data (i.e., standardization of both y_sim_ and y_obs_) that uses information about the target series. Thus, the NSE conservatively uses information about the target’s mean to penalize the measure of a simulation’s performance, while r_p_ optimistically uses similar information to effectively augment the simulation when assessing its performance.

### 2.3. CN runoff correction modeling

As shown in the results below, the high values of r_p_ for the average catchment in most sections along with the low NSE values for the same catchments could indicate that the NDVI CN runoff series were not very close to the NLDAS runoff series, but that nonetheless the two series contained much of the same information. This situation is analogous to comparing measurements taken in the wrong units (e.g., Celsius vs Fahrenheit). Accordingly, we developed correction models to investigate if we could reliably correct the NDVI-CN runoff time series using NLDAS runoff as the target. Catchments with fewer than 100 NDVI-CN event days were excluded from the analysis to ensure sufficient data across all three temporal splits.

We use the Python (version 3.8) programming language to develop software implementing a modeling framework that would allow for a flexible and repeatable analysis of a variety of approaches for generating a set of one or more NDVI-CN correction models. Because our database includes runoff time series for numerous locations characterized as catchments, sections, provinces, and divisions, we allow for distinct models to be estimated for each item in a geographic grouping or level (e.g., one CONUS model or eight physiographic division models, etc.); we refer to this as the *geographic modeling scope*. Additionally, we allow for models to estimate runoff corrections conditional on a different, finer geographic level (e.g., physiographic section within a model of a single physiographic province, or a physiographic province within a model of a single physiographic domain, etc.); we refer to this as the *geographic modeling level*. In the results below, we set *geographic modeling scope* to physiographic division and we set the *geographic modeling level* to physiographic section.

We develop correction models of NDVI-CN, y_CN_ to predict the NLDAS target runoff values, y_NLDAS_, according to,

$$y_{\text{NLDAS},t,g}=f_{G}\left(y_{CN,t,g},g\right)+e_{CN,t,g}$$

where *t* indexes time in days, *G* indexes the *geographic modeling scope* of the transformation *f*, *g* indexes the *geographic modeling level* (i.e., *physiographic section*) associated with each runoff value, and *e*_*CN,t,g*_ is the error. For the correction function, *f*, we develop the option for employing several statistical regression techniques utilizing *pipelines*, *transformers*, and *estimators* from Python’s Scikit-Learn (version 0.24.1) machine learning package (Pedregosa et al., 2011). *Pipelines* are a sequence of data *transformers* and statistical *estimators* that can be *fit* to training data to estimate a model; that model can then be used to *predict* with potentially different data. Pipelines always end with an estimator (e.g., linear regression) and may include data transformation steps such as for standardization and creation of interaction and polynomial terms. Conveniently, transformations such as standardization that are estimated during model training are stored for use with future predictions.

For this paper, we developed pipelines implementing the following statistical estimators: OLS linear regression (lin-reg), lasso regularized linear regression (lasso), ridge regularized linear regression (ridge), elastic-net regularized linear regression (elastic-net), and a gradient boosting regression tree ensemble (GBR). Each of the pipelines is preceded by a global step where the *geographic modeling level* is used to create a set of binary dummy variables identifying the geographic membership of each runoff value. When creating dummy variables, no values were dropped from each level, so no constant term was included as a regressor. This approach avoids perfect-multicollinearity and allows for interpretation of coefficients on dummy variables that does not rely on comparison to an excluded category. Each of the four linear regression pipelines include polynomial terms for the continuous NDVI-CN runoff series interacted with each of the geographic modeling level dummy variables. To avoid perfect multicollinearity, these polynomial terms are not included as non-interacted standalone variables. We also developed the option to use nested cross-validation to choose the optimal polynomial degree for each model, though we did not use that setting in the results presented in this paper in favor of a more geographically specific model selection approach discussed below.

To maintain the integrity of pipelines, we used Python objects to create wrappers that use multi-indexed Pandas *dataframes* (McKinney 2011) to retain information about the ComID associated with each corrected or uncorrected runoff value. This extra programming step was helpful for maintaining data integrity because the scikit-learn estimators and transformers used in the pipelines utilize Numpy array objects (Pedregosa et al., 2011; Harris et al., 2020). When used for cross-validation, each pipeline wrapper also automatically checks for, logs, and removes any catchment IDs (ComIDs) used at training, to guard against data leakage, which could lead to downward biased prediction error estimates and incorrect inference in the model selection process.

When creating a correction model, we have the opportunity for dividing up the data into different models for different values of uncorrected runoff. For example, because the CN method tends to predict no runoff for days with low values of precipitation, the optimal correction model for those values is likely quite different than for days when precipitation is high. For this paper we only develop the capability of splitting uncorrected NDVI-CN runoff values based on whether they are equal to zero, but it would be simple to add other approaches such as a quantile-based split. The split we chose makes sense particularly because zero and nonzero runoff values have distinct data generating processes due to the event-based nature of the CN method.

In our simple point-to-point correction method, there are two straightforward approaches to correcting zero runoff predictions from the NDVI-CN model. First, we consider the mean of observed runoff, conditional on the geographic modeling level, and use that value as the correction. Second, we consider an otherwise identical model that retains the uncorrected value of zero, labeled as *flat0* in the results below. The positive NDVI-CN values are used to train the pipeline, creating a model that can be used for prediction. At the time of prediction, NDVI-CN values are divided into zero and nonzero rows, fed into the appropriate model, and reassembled into a *dataframe* with each row indexed by ComID and date.

Each of the pipelines we created present several opportunities for hyper-parameter tuning. We develop the option to use nested cross-validation for assessing the predictive performance of all hyper-parameter combinations using Scikit Learn’s optimized nested cross validation estimators (e.g., *LassoCV* instead of *Lasso*) when available and the Scikit Learn nested GridSearchCV tool otherwise. These nested cross-validation estimators utilize repeated k-fold cross-validation on the training data passed when fitting a pipeline (which itself may be part of a broader cross-validation assessment). The tool chooses the combination of hyperparameters that has the highest average test R^2^ (i.e., NSE) across inner cross-validation folds, and refits the pipeline using those values on the training data passed to the estimator. Fig. 2 illustrates the nested-cross validation process for a single leave-one-catchment-out split for a single physiographic section. The four ComIDs in each split of nested cross-validation in Fig. 2 correspond to the 4 of 5 catchments for used for training in Fig. 1. The nested cross validation approach to hyper-parameter tuning is computationally intensive at the time of fitting a model.

Because the *geographic modeling scope* can be broader than the *geographic modeling level*, it may be the case that the best combination of hyper-parameters varies from one location/level to the next. To allow for flexible hyper-parameter selection across levels, we developed the option for running and selecting from multiple pipelines with varying hyper-parameter values. This is an alternative to tuning the hyper-parameter values through nested cross-validation in a single pipeline. This alternative approach to handling hyper-parameter values leads to a much longer list of models to select from. In comparison to hyper-parameter tuning with nested cross-validation, this approach is less computationally intensive at the time of fitting the models and more computationally intensive at the time of testing the models.

In the OLS linear regression and regularized linear regression models presented in the results below, we created separate pipelines for each maximum polynomial degree from one to five. We used nested cross-validation to choose hyper-parameters for regularization strength in each pipeline and this particular division balances the computational efficiency of the Scikit-Learn cross validated regularization estimators (e.g., *LassoCV*) and the flexibility of separate models for each maximum polynomial degree.

While double cross validation can produce an *approximately unbiased* estimate of prediction error, choosing from many models the one that seems to have the best prediction error can potentially lead to an optimistic estimate of prediction error. Because we have eighteen years of data in our runoff database, we developed and used in the results below the option for using a separate validation set for reporting the final model accuracy. As discussed in the Introduction, we use the term *validation* to describe the final assessment of the accuracy of the selected model.

Once the cross-validation assessment is complete for each pipeline, all results are compared and the software selects for each location in the *geographic modeling level* the pipeline with the best average leave-one-catchment-out cross-validation NSE over the test data. The uncorrected NDVI-CN runoff series is also considered as a candidate in the model selection process. There is no temporal or spatial cross-validation necessary for the NDVI-CN series due to the lack of a correction model, but to ensure comparability, accuracy is assessed on the same chronological split of test data.

After model selection and model validation are complete, the statistical pipelines associated with each selected model are refit using all of the data in the runoff database to obtain a final model for production use. The resulting model has not been validated in a strict sense, but the modeling approach, as implemented in the statistical pipeline has been validated. Additionally, due to the larger training dataset, the refit model is likely to have less bias and variance than the sub-models estimated during the cross-validation experiment that informs our accuracy assessment presented below.

## 3. Results and discussion

### 3.1. NDVI-CN runoff

**Fig. 3.**
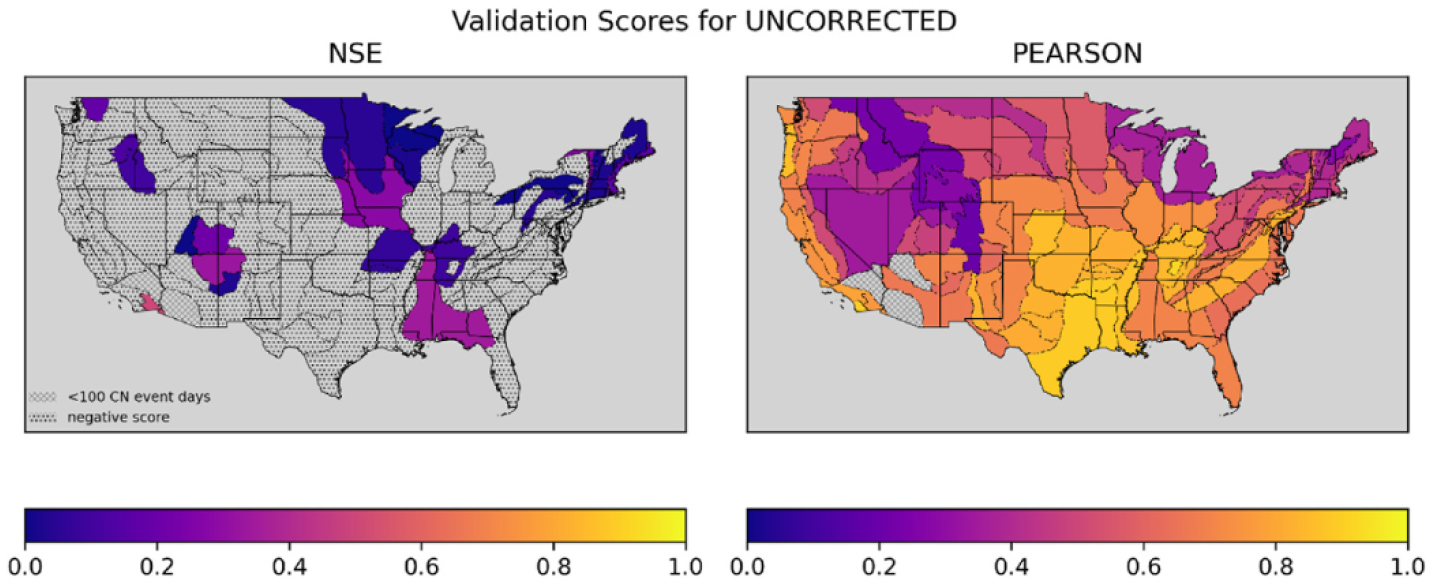
Two maps of CONUS with Physiographic Sections colored to indicate NSE and Pearson’s Correlation Coefficient for NDVI-CN generated runoff over the validation split.

To better understand geographic variability in the accuracy of the uncorrected NDVI-CN runoff time series relative to the NLDAS runoff time series, we calculated NSE and r_p_ for each catchment. Then we grouped the sampled catchments by physiographic section and averaged each accuracy measure within each section. The resulting maps can be seen in Fig. 3. Because we are not interested in physiographic sections where the model performs worse than a simple average, we censored locations with negative average accuracy metrics when shading the maps. This leaves more room in the map’s polygon fill gradient to facilitate interpretation for physiographic sections where the simulation has some credibility (i.e., NSE>0). We also use identical scaling of the fill-gradient to the accuracy metrics across all maps in this paper to facilitate comparisons among figures. Notably, the GLDAS accuracy maps in Appendix 1 share their own, distinct scaling.

Fig. 3 shows overwhelmingly higher values for the averaged r_p_ relative to averaged NSE. The juxtaposition of high r_p_ and low NSE for the same simulated and observed runoff values can be explained by at least two possibilities:

1. Physiographic sections contain catchments that perform very differently across the models estimated during the leave-one-catchment-out cross-validation experiment. One large, negative NSE value for a single catchment in a section can dominate the average value of NSE. For r_p_, a poor prediction can’t be below minus one, so a single poorly performing catchment cannot dominate the average r_p_.
2. The NDVI-CN runoff simulations correlate with the NLDAS runoff values similarly across catchments in a physiographic section, but the NDVI-CN values suffer from a scaling problem. This explanation hints at a possibility of correcting this scaling problem to obtain precise automated CN generated runoff predictions.

### 3.2. CN runoff correction modeling

**Fig. 4.**
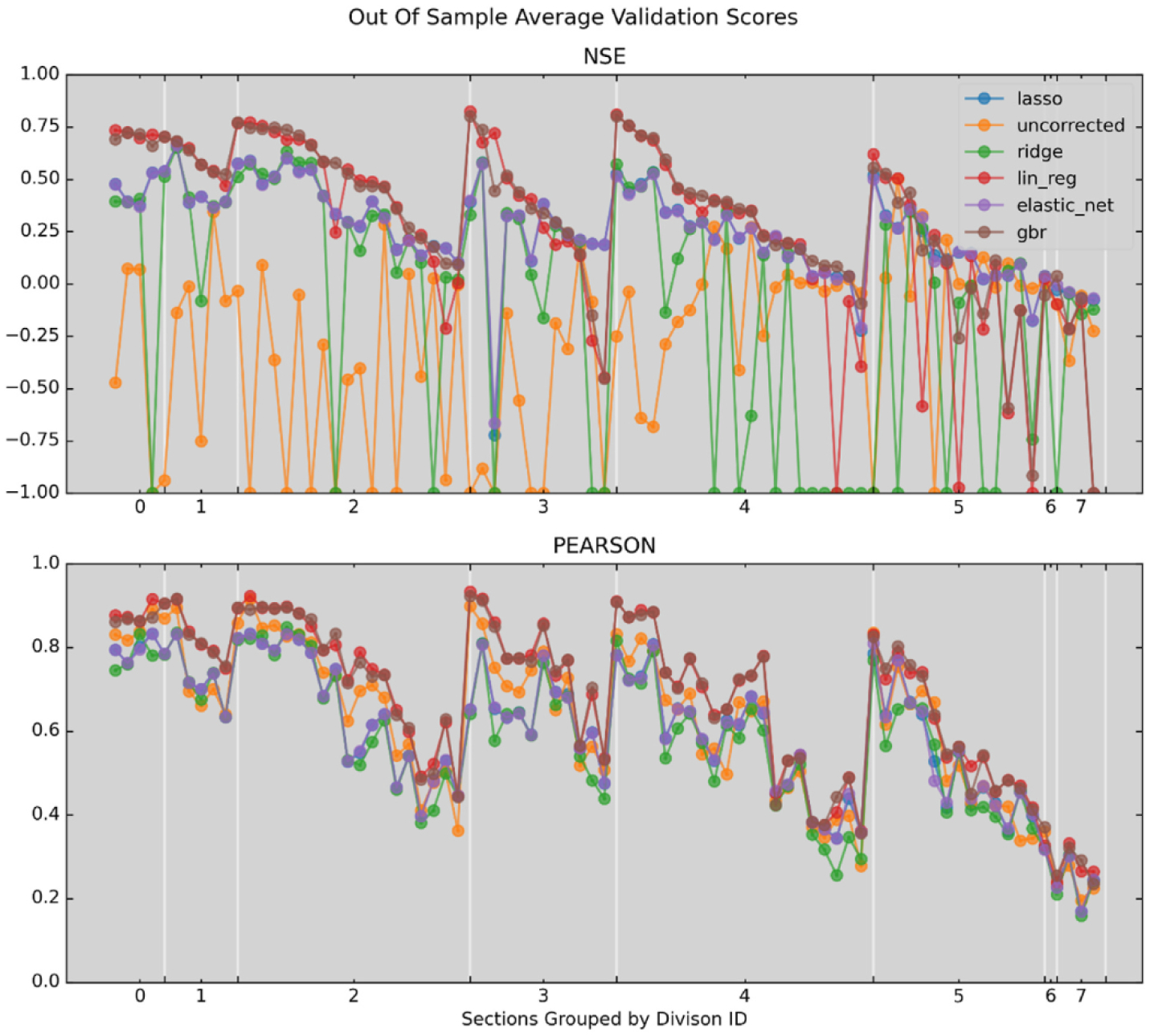
Two plots of model accuracy scores (truncated at −1) with physiographic sections sorted by NSE and grouped in physiographic domains sorted by average NSE.

For this paper we estimate correction models using physiographic domain as the *geographic modeling scope* and physiographic section as the *geographic modeling level*. A visual comparison of the validation average NSE scores of the leave-one-catchment-out cross-validation assessment for each pipeline are presented in Fig. 4. In this figure, physiographic domains are arranged in order of decreasing NSE (averaged across sections) and sections are arranged in order of decreasing NSE (averaged across catchments). Each point for each statistical estimator is the validation NSE of the best performing model of that type, where selection is based on the test data not validation data. The numbering of the sorted physiographic domains can be found in Table 2 along with the validation NSE, and model details for the model that scored highest on the test data.

In Fig. 4, one of the more remarkable patterns is that there appears to be no relationship between the uncorrected NSE and the corrected NSE. This indicates that NDVI-CN generated runoff time series diverge from the NLDAS runoff time series quite differently across sections even in the same domain. Fig. 4 is also useful for assessing the relative strengths of the different statistical techniques. From a machine learning perspective, it is interesting that the regularized regression methods are frequently dominated by the OLS linear regression models. A finer spaced grid of regularization hyper-parameters that include smaller values for regularization strength may lead to improved performance. However, regularization is intended to reduce over-fitting, and the large number of observations and relatively few parameters in the models may prevent over-fitting without regularization penalties.

**Fig. 5.**
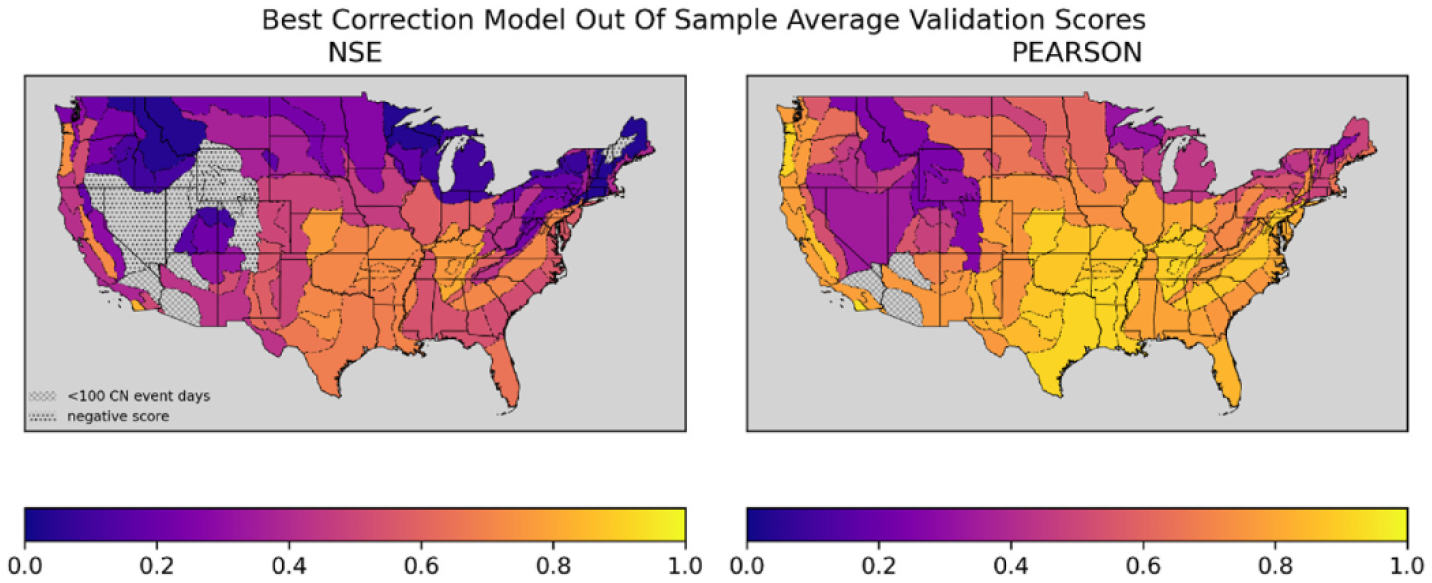
Two maps of CONUS with Physiographic Sections colored to indicate NSE and Pearson’s Correlation Coefficient for each section’s best correction model generated runoff over the validation split.

**Fig. 6.**
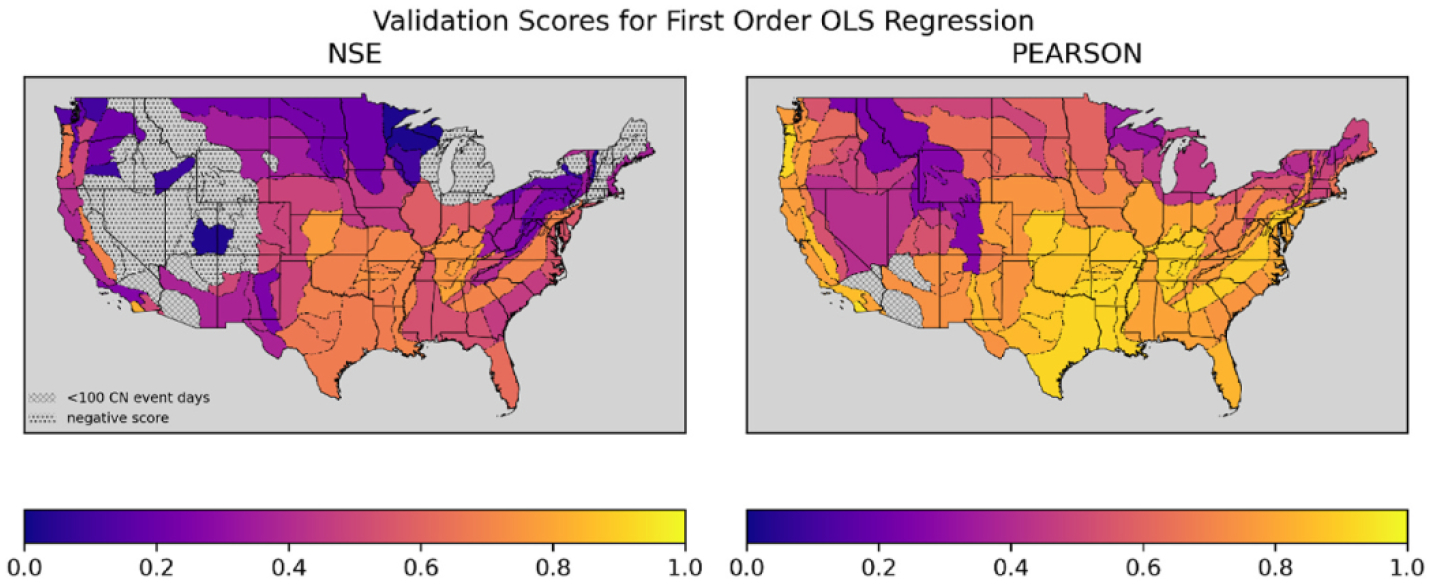
Two maps of CONUS with Physiographic Sections colored to indicate NSE and Pearson’s Correlation Coefficient for first order linear regression correction generated runoff over the validation split.

Fig. 5 shows the validation scores for the selected estimator for each physiographic section. The NSE results provide the best indication of the likely accuracy of future runoff simulations from the models considered in this analysis. In this map, r_p_ has visibly increased relative to the same measure for uncorrected NDVI-CN runoff from Fig. 3, suggesting accuracy improvements come partially from the estimator learning the NLDAS series beyond just learning how to rescale the uncorrected NDVI-CN runoff values. The r_p_ values can rise when a model with a non-linear transformation (including higher than first degree polynomials) is selected or because of the improvement in correlation from the correction for NDVI-CN non-event predictions of zero. By considering the patterns in r_p_ in Fig. 6 relative to Fig. 3 (both of which benefit from the rescaling inherent to r_p_), the improvement from the transformation of nonzero runoff values can be distinguished from improvements that come from correcting the non-event values from zero to the mean of the zero-runoff-days in the training data. The similarity in r_p_ across the correction models suggest a substantial bulk of improvement in r_p_ is due to the non-event, zero runoff corrections.

By comparing the NSE scores for the best correction models in Fig. 5 with the NSE scores from the first order linear regression models in Fig. 6 and the original NSE scores for the uncorrected NDVI-CN runoff series in Fig. 3 we can see that the bulk of the improvement in accuracy in the correction models is attainable with a simple linear rescaling of the nonzero, event values and a simple shift of the non-event, zero values. It is useful to compare the values of r_p_ in Fig. 3 to the corrected NSE values shown in Fig. 6. The r_p_ benefits from an in-sample transformation to account for differences in the mean of each series, an inherent part of the r_p_ metric. In contrast, the coefficients from the first order linear regression correction models also implement a linear transformation, but from out of sample (relative to the testing and validation splits over which the metrics are calculated) training data. The r_p_ is also not squared like NSE, but otherwise the measures are similar and comparing them indicates how well a simple linear correction can generalize to leverage the available information to correct linear scaling problems for future predictions.

From visual inspection of the accuracy maps, it appears physiographic sections with more snowfall or less precipitation tend to perform poorly across the various runoff simulations we conducted. Because none of the models we developed for this paper use information about time for training or prediction, simple augmentations like monthly or bi-weekly time dummy variables may increase runoff accuracy, particularly in snowy areas with consistent annual runoff patterns.

**Fig. 7.**
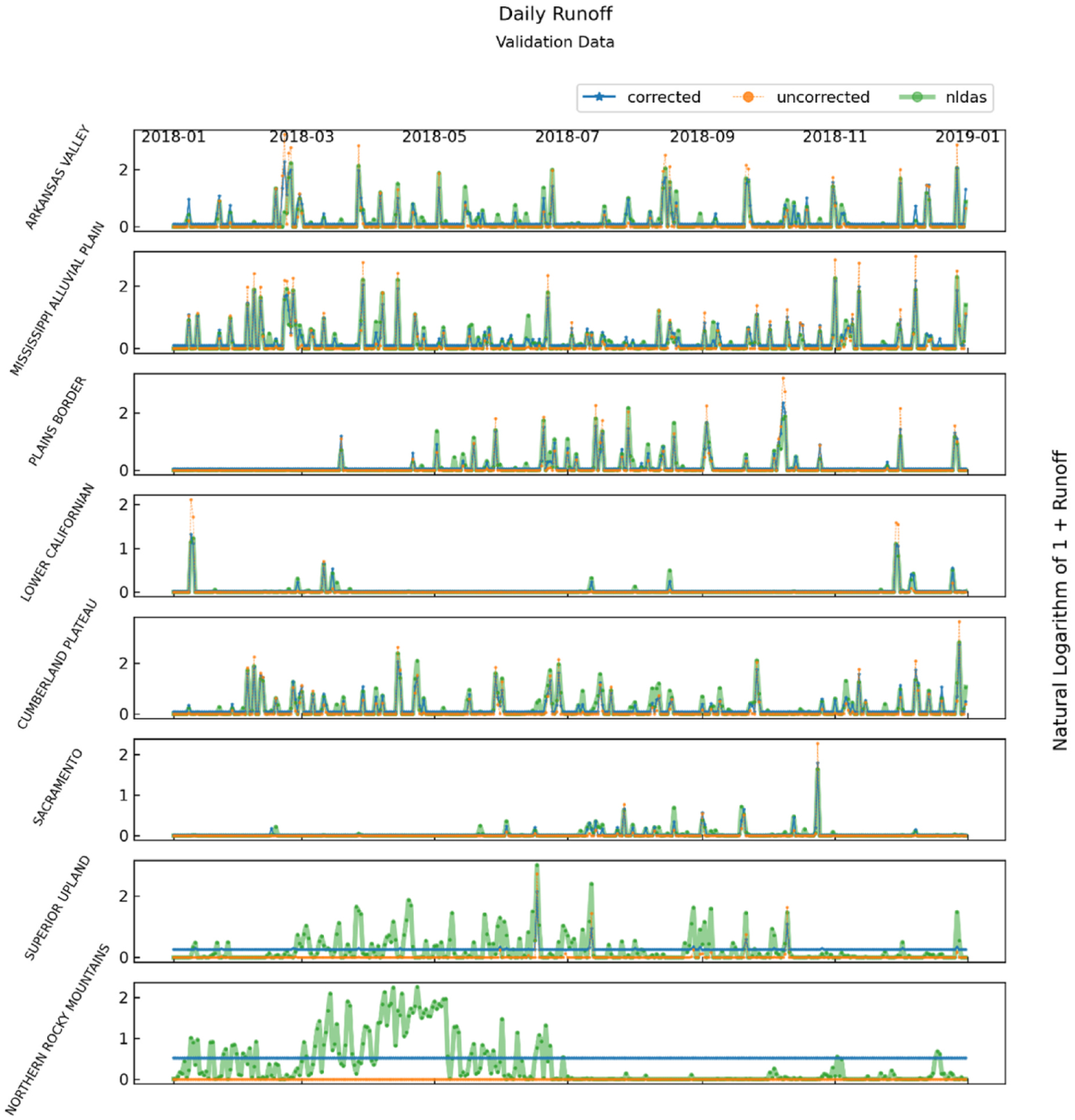
One year of NLDAS, NDVI-CN, and best correction generated runoff for the physiographic section in each division with the highest corrected validation NSE.

Fig. 7 contains runoff and simulation data for the last year of the validation split for the physiographic section in each physiographic domain with the best corrected runoff NSE. Each of these sections is the first section in each division in Table 2 and in Fig. 4. Each pane includes the following three runoff series: uncorrected NDVI-CN generated, best correction model generated, and NLDAS generated. For all runoff time series plots in this paper, the runoff values on the vertical axes are transformed by taking the natural logarithm of one plus runoff. The logarithmic transformation makes it easier to see patterns at both low and high values of runoff and adding 1 prior to the logarithmic transformation keeps runoff values of zero at zero.

Fig. 7 is useful for developing an understanding of the overall behavior of the runoff series across the diverse physiographic domains. The impact of poor event detection is particularly visible in the bottom two series where the nonzero correction model predictions are markedly above zero. It is also readily apparent from several panes that the correction models reduce the frequency of dramatic runoff over-predictions generated by NDVI-CN.

**Fig. 8.**
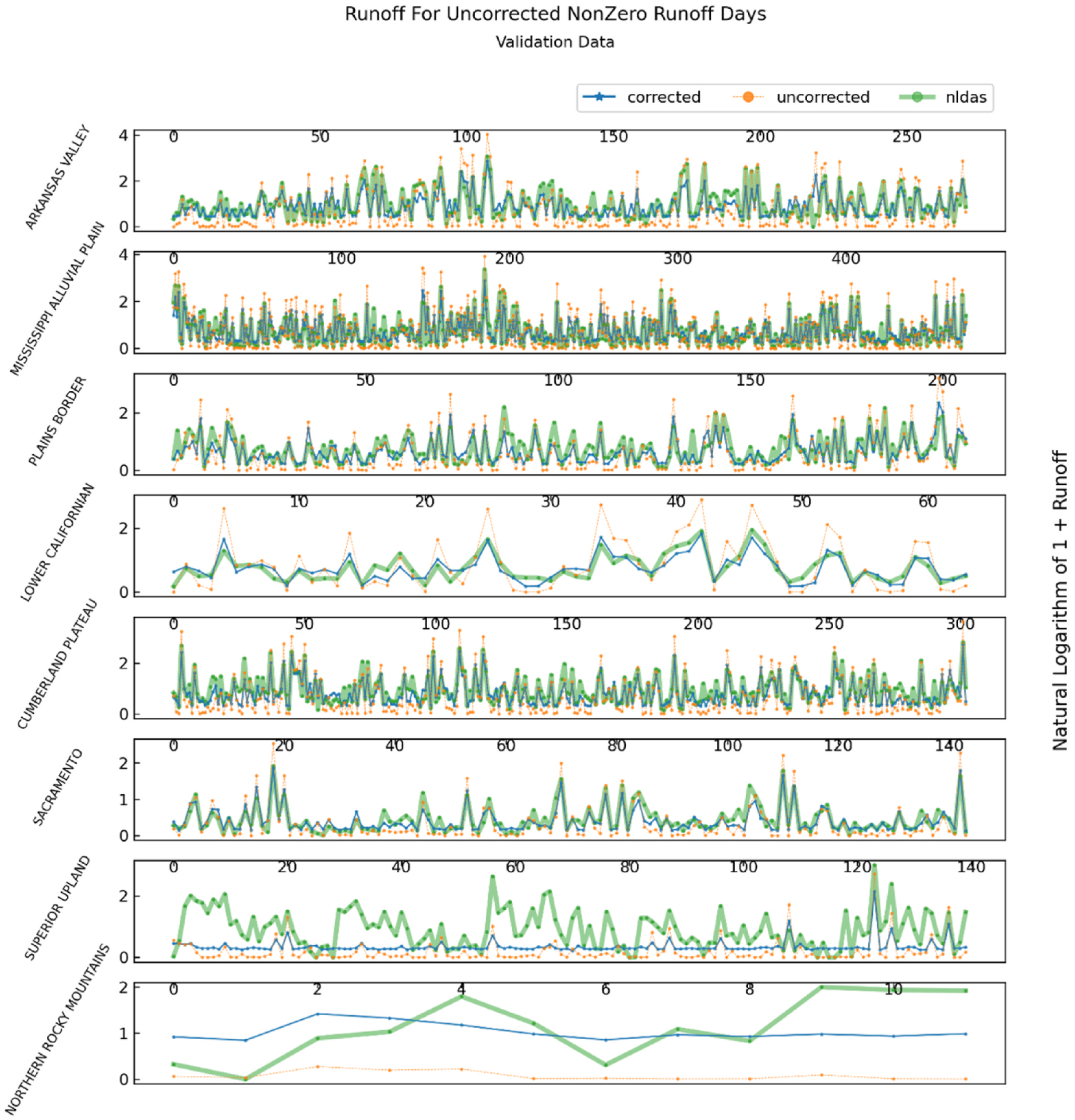
Nonzero CN event days during the validation time split for NLDAS, NDVI-CN, and best correction generated runoff for the physiographic section in each division with the highest corrected validation NSE.

**Fig. 9.**
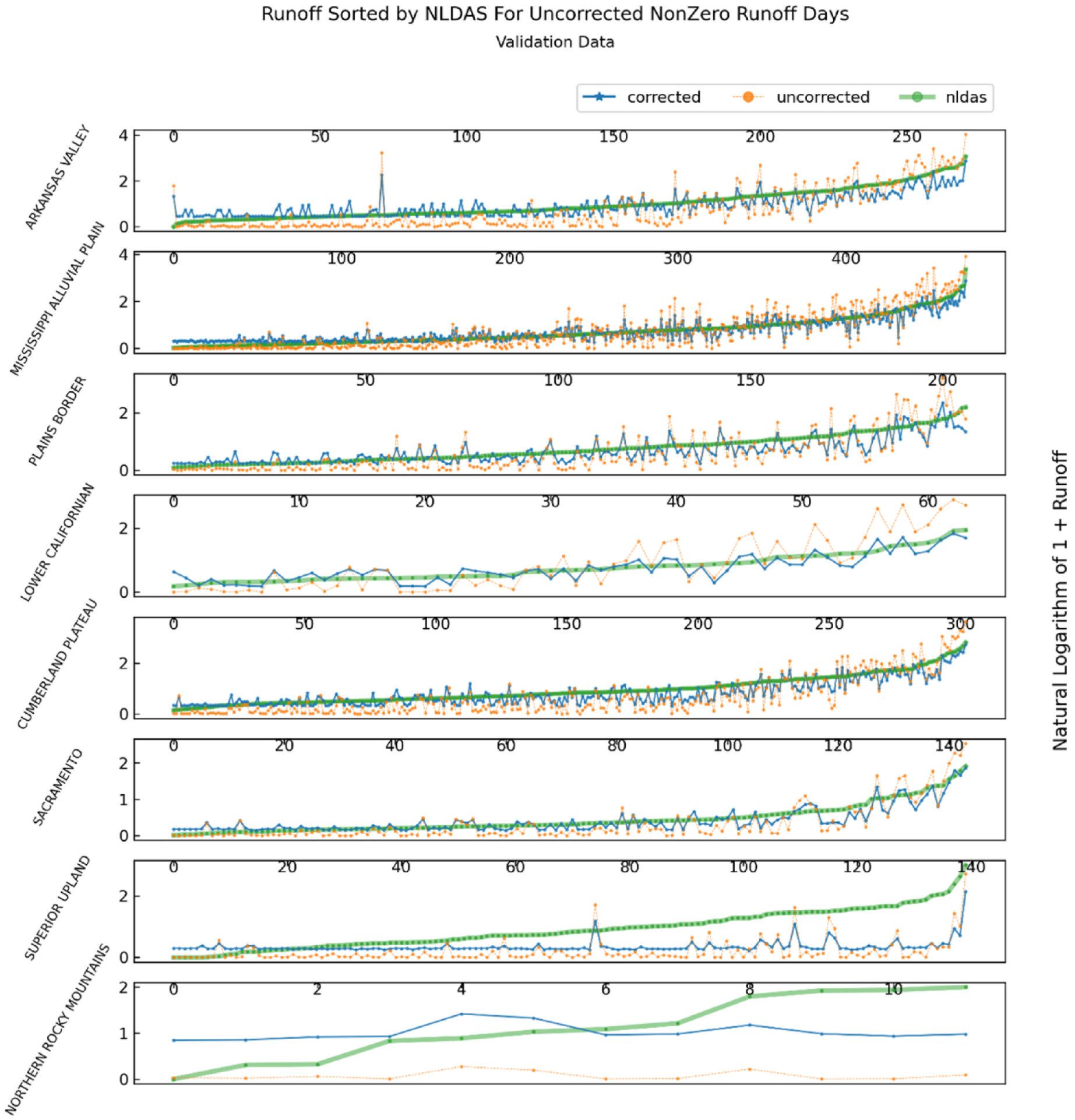
Nonzero CN event days during the validation time split for NLDAS, NDVI-CN, and best correction generated runoff, sorted by NLDAS runoff for the physiographic section in each division with the highest corrected validation NSE.

To better distinguish between runoff performance during NDVI-CN nonzero runoff events, we created separate runoff plots spanning the validation temporal split for NDVI-CN events with nonzero uncorrected simulated runoff and NDVI-CN non-events with zero uncorrected simulated runoff. Fig. 8 shows only the event days when NDVI-CN predicts positive runoff. Across the top of each pane in the figure is an index that numbers the days of nonzero NDVI-CN runoff in each physiographic division. It is helpful to consider these same nonzero runoff values, but ordered by ascending observed runoff (i.e., NLDAS runoff), as illustrated in Fig. 9. Here the reader can see patterns of under or overprediction of the final corrected runoff models, which can be used to validate or invalidate the use of these models for various real-world decision-making applications.

It is interesting to compare the runoff predictions for the Mississippi Alluvial Plain (MAP) and the Arkansas Valley (AV) (the top 2 panes in Fig. 9. As can be seen in Table 2, both sections have high NSE values, and the selected model for the MAP is a first order linear regression, while the AV correction is a 3rd order linear regression. For the days in the AV with the highest runoff, the polynomial correction appears to help achieve a close fit, while for the MAP the corrected runoff values seem to have a downward bias when observed runoff is highest. The AV has nearly twice as many observations, which may be important for estimating higher order polynomial terms with sufficient precision to enhance predictive accuracy over simpler models.

As indicated by Table 2, the Plains Border section uses GBR as the selected correction estimator. A close examination of the differences between the uncorrected runoff values and corrected runoff values in Fig. 9 reveals several instances of non-monotonic transformations, where NDVI-CN runoff falls and corrected runoff falls but then rises. This can be seen around day 80, for example. In the same figure, the Lower Californian section, with relatively few NDVI-CN nonzero event days, shows a marked improvement in accuracy at high runoff values while reducing the variability of NDVI-CN runoff. This last pattern, a reduction in corrected runoff variability relative to NCVI-CN variability is the most discernible feature of the first five panes in Fig. 9.

**Fig. 10.**
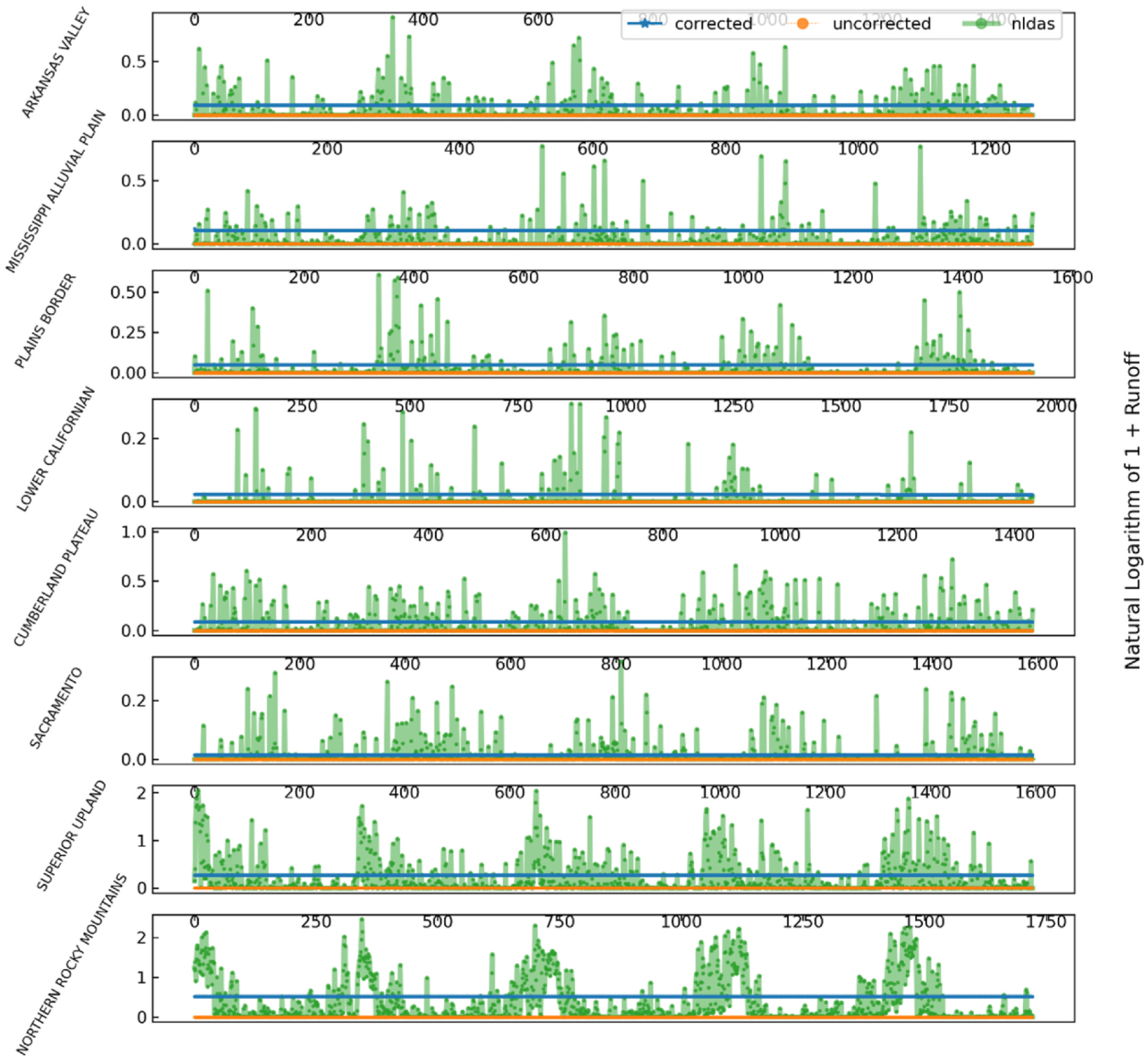
Zero runoff, CN non-event days during the validation time split for NLDAS, NDVI-CN, and best correction generated runoff for the physiographic section in each division with the highest corrected validation NSE.

To better understand the days when NDVI-CN predicts zero runoff, we also developed Fig. 10 showing the NDVI-CN zero runoff days in the validation split. It is important to note that the vertical scale on these graphs varies widely. The NDVI-CN method fails to detect substantial runoff events in a seasonal pattern in NLDAS runoff in the Superior Upland and Northern Rocky Mountains physiographic sections. There also are likely statistically significant seasonal patterns in the NDVI-CN zero runoff days that could be addressed by adding additional complexity to the zero-runoff correction models.

While the validation and estimation framework we used for developing the NDVI-CN correction models is complex, the underlying models are relatively simple because they lack an awareness of time. Broadening the information set available for prediction in both the zero-runoff and nonzero-runoff models to include past time periods would potentially overcome limitations associated with the event-based nature of NDVI-CN. Including precipitation and lagged precipitation similarly would likely provide opportunities for increasing model skill. Variables for seasonality or more sophisticated time series approaches such as wavelets also would likely increase model accuracy, particularly for locations with substantial snow melt and accumulation. However, in the context of the curve number methodology, a simple and effective linear correction is particularly appealing.

In these results we considered only a narrow range of the possibilities for grouping the data by selecting a broad *geographic modeling scope* (physiographic division), and a narrow geographic modeling level (*physiographic section*). At the same time, we used a short list of explanatory variables, so the structure of the matrix of regressors is block diagonal, and thus relatively little information is shared between physiographic sections in each physiographic division for use by the statistical estimators. More complex structures such as overlapping dummy variables (Lim and Hastie 2015) and geographic regressors such as those in StreamCat may help identify stronger patterns in the data. A finer selection for the *geographic modeling scope* would also potentially improve model accuracy by reducing the tendency to lump together *physiographic sections* or more generally *slices* with contrasting snow fall/melt patterns.

## 4. Conclusions

By developing and applying a carefully designed model estimation, selection, and accuracy assessment framework, we have developed correction models to enhance NDVI-CN rainfall-runoff model. The result of our accuracy assessment is a set of validation NSE values that can be used by practitioners who need runoff time series estimates to appropriately curate their data sources and quantify sources of error in downstream modeling applications.

Because the curve number approach to runoff modeling is one of the simplest and least data intensive approaches, it is fascinating that runoff estimated using a somewhat inflexible, automated approach to quantifying hydrologic condition (i.e., NDVI-CN) has such high linear correlation with an ensemble of state-of-the-art LSMs. Further, for much of the country, this relationship is stable and can be leveraged into simple first order linear regression correction models with skillful predictions, as judged by NSE and illustrated in Fig. 6.

During this research, we became aware of the importance of including a simple metric like r_p_ to contrast more stringent accuracy measures like NSE or KGE. While time series plots of simulated and observed runoff can help the analyst spot information patterns that can be leveraged to build correction models, it is useful to have a metric that quantifies these patterns. Because r_p_, R^2^, and NSE have so much in common, there may be a need for a generalized version of r_p_ in the same manner that KGE generalizes NSE. Non-linear correlation measures like Spearman’s rank order correlation coefficient may also be useful for spotting non-linear patterns in the data.

## Supplementary Material

Supplement1

## Figures and Tables

**Table 1:** NDVI hydrologic condition table.

Land Cover Class	Poor	Normal	Good
41 - Deciduous Forest	NDVI <6500	6500 ≤ NDVI ≤ 7500	NDVI >7500
42 - Evergreen Forest	NDVI <6500	6500 ≤ NDVI ≤ 7500	NDVI >7500
43 - Mixed Forest	NDVI <6500	6500 ≤ NDVI ≤ 7500	NDVI >7500
52 - Shrub/Scrub	NDVI <5500	5500 ≤ NDVI ≤ 6500	NDVI >6500
71 - Grassland/Herbaceous	NDVI <5000	5000 ≤ NDVI ≤ 6000	NDVI >6000
81 - Pasture/Hay	NDVI <5000	5000 ≤ NDVI ≤ 6000	NDVI >6000
82 - Cultivated Crops	NDVI <4000	4000 ≤ NDVI ≤ 5000	NDVI >5000

**Table 2:** Estimator selection and validation score by physiographic section.

Division ID	Division	Province	Section	Selected Estimator	NSE
0	Interior Highlands	Ouachita	Arkansas Valley	LIN_REG-1^a^	0.735
		Ozark Plateaus	Springfield-Salem Plateaus	LIN_REG-5	0.725
			Boston “Mountains”	GBR-200_0.1_0.8_2^b^	0.716
		Ouachita	Ouachita Mountains	LIN_REG-1	0.713
1	Atlantic Plain	Coastal Plain	Mississippi Alluvial Plain	LIN_REG-3-FLAT0^c^	0.704
			West Gulf Coastal Plain	GBR-100_0.05_0.6_2-FLAT0	0.682
			Floridian	LIN_REG-3	0.650
			Embayed	LIN_REG-2	0.570
			East Gulf Coastal Plain	LIN_REG-3	0.540
			Sea Island	GBR-100_0.05_0.6_2	0.524
2	Interior Plains	Great Plains	Plains Border	GBR-200_0.05_0.8_2-FLAT0	0.773
		Interior Low Plateaus	Nashville Basin	LIN_REG-1	0.773
			Highland Rim	LIN_REG-5	0.756
			Lexington Plain	GBR-200_0.05_0.6_2-FLAT0	0.749
		Great Plains	Central Texas	GBR-100_0.05_0.6_2	0.736
		Central Lowland	Osage Plains	GBR-100_0.05_0.6_2-FLAT0	0.711
		Great Plains	Edwards Plateau	LIN_REG-1-FLAT0	0.666
		Central Lowland	Till Plains	GBR-200_0.05_0.6_2	0.584
		Great Plains	Pecos Valley	GBR-100_0.05_0.8_2	0.579
			Raton	LIN_REG-5	0.547
			Colorado Piedmont	LIN_REG-3-FLAT0	0.502
			High Plains	LIN_REG-1	0.489
		Central Lowland	Dissected Till Plains	LIN_REG-2	0.467
		Great Plains	Missouri Plateau, Unglaciated	LIN_REG-4	0.369
		Central Lowland	Western Lake	GBR-100_0.05_0.8_2	0.270
		Great Plains	Missouri Plateau, Glaciated	LIN_REG-5	0.234
		Central Lowland	Wisconsin Driftless	LASSO-5	0.183
		Great Plains	Black Hills	LASSO-2-FLATO	0.171
		Central Lowland	Eastern Lake	ELASTIC_NET-3	0.103
3	Pacific Mountain System	Lower Californian	Lower Californian	LIN_REG-1-FLAT0	0.824
		Pacific Border	Oregon Coast Range	GBR-100_0.05_0.8_2	0.738
			California Trough	LIN_REG-3	0.719
		Cascade-Sierra Mountains	Middle Cascade Mountains	GBR-100_0.1_0.8_2	0.517
		Pacific Border	Klamath Mountains	GBR-100_0.1_0.6_2-FLAT0	0.464
			California Coast Ranges	LIN_REG-5-FLAT0	0.419
			Los Angeles Ranges	LASSO-1-FLAT0	0.383
		Cascade-Sierra Mountains	Southern Cascade Mountains	GBR-100_0.05_0.8_2-FLAT0	0.300
		Pacific Border	Puget Trough	GBR-100_0.05_0.8_2	0.295
		Cascade-Sierra Mountains	Sierra Nevada	LIN_REG-1-FLAT0	0.294
		Pacific Border	Olympic Mountains	GBR-100_0.05_0.8_2	0.244
		Cascade-Sierra Mountains	Northern Cascade Mountains	RIDGE-1-FLAT0	0.212
4	Appalachian Highlands	Appalachian Plateaus	Cumberland Plateau	LIN_REG-4	0.809
			Cumberland Mountain	GBR-100_0.1_0.8_2	0.759
		Piedmont	Piedmont Upland	GBR-100_0.05_0.6_2	0.710
			Piedmont Lowlands	GBR-200_0.05_0.6_2	0.698
		Valley and Ridge	Tennessee	GBR-100_0.1_0.6_2	0.596
		Blue Ridge	Northern	GBR-200_0.05_0.8_2	0.459
			Southern	GBR-200_0.05_0.8_2	0.435
		Appalachian Plateaus	Kanawha	GBR-200_0.05_0.6_2	0.422
		St. Lawrence Valley	Champlain	LIN_REG-1	0.402
		New England	Seaboard Lowland	GBR-100_0.05_0.6_2	0.394
		Valley and Ridge	Hudson Valley	GBR-100_0.05_0.6_2	0.359
		New England	Taconic	LIN_REG-2	0.352
		Valley and Ridge	Middle	LIN_REG-2	0.233
		Appalachian Plateaus	Catskill	ELASTIC_NET-1	0.229
			Allegheny Mountain	GBR-100_0.05_0.8_8	0.197
			Southern New York	LIN_REG-1	0.190
		New England	Green Mountain	GBR-100_0.1_0.8_2	0.110
		Appalachian Plateaus	Mohawk	GBR-200_0.1_0.6_4	0.087
		Adirondack	Adirondack	GBR-200_0.1_0.6_2	0.084
		New England	New England Upland	GBR-100_0.05_0.8_2	0.038
			White Mountain	UNCORRECTED	−0.043
5	Intermontane Plateaus	Basin and Range	Sacramento	LIN_REG-2	0.620
		Colorado Plateaus	Datil	GBR-100_0.1_0.8_4	0.527
		Basin and Range	Salton Trough	LIN_REG-1	0.505
			Mexican Highland	GBR-100_0.05_0.6_2	0.437
		Colorado Plateaus	Navajo	UNCORRECTED	0.331
		Columbia Plateau	Walla Walla Plateau	LIN_REG-2-FLAT0	0.239
		Colorado Plateaus	Canyon Lands	UNCORRECTED	0.212
		Columbia Plateau	Blue Mountain	GBR-100_0.05_0.6_2-FLAT0	0.157
		Colorado Plateaus	High Plateaus Of Utah	ELASTIC_NET-5-FLATO	0.155
		Columbia Plateau	Harney	LASSO-3	0.149
			Payette	GBR-100_0.05_0.6_2-FLAT0	0.136
			Snake River Plain	GBR-100_0.05_0.8_2	0.113
		Colorado Plateaus	Uinta Basin	RIDGE-3-FLAT0	0.103
		Basin and Range	Great Basin	UNCORRECTED	−0.021
6	Laurentian Upland	Superior Upland	Superior Upland	LASSO-1	0.039
7	Rocky Mountain System	Northern Rocky Mountains	Northern Rocky Mountains	GBR-100_0.1_0.8_2	0.038
		Southern Rocky Mountains	Southern Rocky Mountains	GBR-100_0.05_0.6_4-FLAT0	−0.008
		Wyoming Basin	Wyoming Basin	RIDGE-2-FLAT0	−0.029
		Middle Rocky Mountains	Middle Rocky Mountains	ELASTIC_NET-2-FLAT0	−0.069

a For linear models the number following the estimator’s name is the maximum polynomial degree used for transforming uncorrected runoff for training and prediction.

b For GBR the trailing numbers indicate the following hyper parameter values: number of estimators in the ensemble, the boosting learning rate, the fraction of the training sample to use for stochastic gradient descent, and the maximum tree depth.

c The suffix FLAT0 indicates that a model predicts zero runoff for NDVI-CN zero runoff days instead of using the mean of observed runoff from zero runoff days in the training data.

## Data Availability

Statement: The code used to develop this paper and the appendix can be found at the following sites: https://github.com/quanted/hms-handler/. Curve Number and NDVI dataset can be found at the following site: ftp://newftp.epa.gov/exposure/CurveNumberNDVI Average curve number dataset for NHDPlus catchments can be found at the following site: https://qed.epa.gov/hms/rest/api/info/catchment?cn=true&comid=COMID where COMID is NHDPlusV2 catchment ID.
