# Supplementary material for: Catchment scale runoff time-series generation and validation using statistical models for the Continental United States
Source: Environ Model Softw. Author manuscript; Available in PMC 2023 Mar 1. (PMC8931853; doi:10.1016/j.envsoft.2022.105321)
Supplement: Supplement1 [file NIHMS1787843-supplement-Supplement1.docx]

**Guide for authors:** [**https://ascelibrary.org/doi/10.1061/9780784479018.ch05**](https://ascelibrary.org/doi/10.1061/9780784479018.ch05)

**Title:** Curve number generated surface runoff time-series correction using statistical models at NHDPlus catchment scale

**Author byline:** Patton, Douglas^1^;Smith, Deron^2^; Muche, Muluken E^3^; Wolfe, Kurt^2^; Parmar, Rajbir^2^; Johnston, John M^2^.

**Affiliations:** ^1^Oakridge Institute of Science and Education; ^2^ Environmental Protection Agency; ^3^Oakridge Institute of Science and Education, National Science Foundation

# Appendix – GLDAS Results

We designed our study and software in a flexible manner, so producing equivalent results using GLDAS rather than NLDAS precipitation and runoff data was a simple extension of our NLDAS results. Generally, GLDAS results are considerably less accurate and there are a few missing values from failed requests or from too few CN events. Table A1 shows the quantitative accuracy results with sorting of physiographic sections and physiographic domains based on these GLDAS results, so ordering is different than in Table 2 in the main paper.

## Figure Captions

**FIg A1.** Two maps of CONUS with Physiographic Sections colored to indicate NSE and Pearson’s Correlation Coefficient for NDVI-CN generated runoff over the validation split.

**Fig. A2.** Two plots of model accuracy scores (truncated at -1) with physiographic sections sorted by NSE and grouped in physiographic domains sorted by average NSE.

**Fig. A3.** Two maps of CONUS with Physiographic Sections colored to indicate NSE and Pearson’s Correlation Coefficient for each section’s best correction model generated runoff over the validation split.

**Fig. A4.** Two maps of CONUS with Physiographic Sections colored to indicate NSE and Pearson’s Correlation Coefficient for first order linear regression correction generated runoff over the validation split.

**Fig. A5.** One year of GLDAS, NDVI-CN, and best correction generated runoff for the physiographic section in each division with the highest corrected validation NSE.

**Fig. A6.** Non-zero CN event days during the validation time split for GLDAS, NDVI-CN, and best correction generated runoff for the physiographic section in each division with the highest corrected validation NSE.

**Fig. A7.** Non-zero CN event days during the validation time split for GLDAS, NDVI-CN, and best correction generated runoff, sorted by GLDAS runoff for the physiographic section in each division with the highest corrected validation NSE.

**Fig. A8.** Zero runoff, CN non-event days during the validation time split for GLDAS, NDVI-CN, and best correction generated runoff for the physiographic section in each division with the highest corrected validation NSE.

**Table A1.** Estimator Selection and Validation Scores by Physiographic Section

| **Division ID** | **Division** | **Province** | **Section** | **Selected Estimator** | **NSE** |
| --- | --- | --- | --- | --- | --- |
| 0 | Interior Highlands | Ozark Plateaus | Boston "Mountains" | LIN_REG-1 | 0.589 |
|  |  |  | Springfield-Salem Plateaus | UNCORRECTED | 0.505 |
|  |  | Ouachita | Arkansas Valley | LIN_REG-1 | 0.483 |
|  |  |  | Ouachita Mountains | RIDGE-1 | 0.175 |
| 1 | Appalachian Highlands | Appalachian Plateaus | Cumberland Plateau | GBR-200_0.05_0.6_4 | 0.738 |
|  |  |  | Cumberland Mountain | LIN_REG-1 | 0.713 |
|  |  | Valley and Ridge | Tennessee | UNCORRECTED | 0.562 |
|  |  | Appalachian Plateaus | Kanawha | GBR-100_0.05_0.6_2 | 0.521 |
|  |  | Piedmont | Piedmont Upland | LIN_REG-1 | 0.339 |
|  |  |  | Piedmont Lowlands | GBR-100_0.05_0.6_4 | 0.334 |
|  |  | Blue Ridge | Northern | GBR-100_0.05_0.6_4 | 0.323 |
|  |  |  | Southern | GBR-100_0.05_0.6_2 | 0.319 |
|  |  | Valley and Ridge | Middle | GBR-100_0.05_0.6_2 | 0.247 |
|  |  | Appalachian Plateaus | Allegheny Mountain | GBR-200_0.1_0.8_2 | 0.230 |
|  |  | St. Lawrence Valley | Champlain | GBR-100_0.05_0.8_2 | 0.080 |
|  |  | Valley and Ridge | Hudson Valley | GBR-200_0.1_0.6_2 | 0.078 |
|  |  | New England | Seaboard Lowland | GBR-100_0.05_0.8_2 | 0.016 |
|  |  | Adirondack | Adirondack | GBR-100_0.05_0.8_2 | 0.009 |
|  |  | New England | Green Mountain | LIN_REG-3 | 0.007 |
|  |  | Appalachian Plateaus | Southern New York | GBR-100_0.05_0.8_2 | 0.000 |
|  |  |  | Catskill | LASSO-1 | -0.001 |
|  |  | New England | Taconic | GBR-100_0.05_0.8_2 | -0.003 |
|  |  |  | White Mountain | GBR-100_0.05_0.8_2 | -0.010 |
|  |  | Appalachian Plateaus | Mohawk | GBR-100_0.05_0.8_2 | -0.019 |
|  |  | New England | New England Upland | GBR-100_0.05_0.8_2 | -0.035 |
| 2 | Intermontane Plateaus | Basin and Range | Salton Trough | LIN_REG-1 | 0.474 |
|  |  |  | Sacramento | ELASTIC_NET-1 | 0.463 |
|  |  |  | Mexican Highland | GBR-100_0.05_0.8_2 | 0.419 |
|  |  | Colorado Plateaus | Datil | LIN_REG-5 | 0.324 |
|  |  |  | Navajo | LASSO-5-FLAT0 | 0.290 |
|  |  |  | Uinta Basin | LIN_REG-3 | 0.232 |
|  |  |  | Canyon Lands | RIDGE-5-FLAT0 | 0.210 |
|  |  | Columbia Plateau | Harney | GBR-100_0.05_0.8_2 | 0.170 |
|  |  |  | Walla Walla Plateau | GBR-100_0.05_0.6_4 | 0.082 |
|  |  |  | Payette | GBR-100_0.05_0.8_2 | 0.075 |
|  |  | Basin and Range | Great Basin | GBR-100_0.05_0.8_2 | 0.047 |
|  |  | Columbia Plateau | Snake River Plain | LASSO-3 | 0.022 |
|  |  |  | Blue Mountain | LIN_REG-1 | -0.002 |
|  |  | Colorado Plateaus | High Plateaus Of Utah | ELASTIC_NET-4-FLAT0 | -1.958 |
| 3 | Laurentian Upland | Superior Upland | Superior Upland | ELASTIC_NET-1 | -0.014 |
| 4 | Rocky Mountain System | Southern Rocky Mountains | Southern Rocky Mountains | GBR-100_0.05_0.6_2-FLAT0 | 0.096 |
|  |  | Wyoming Basin | Wyoming Basin | GBR-100_0.05_0.6_2 | 0.022 |
|  |  | Northern Rocky Mountains | Northern Rocky Mountains | LASSO-1-FLAT0 | -0.061 |
|  |  | Middle Rocky Mountains | Middle Rocky Mountains | GBR-100_0.05_0.6_2-FLAT0 | -0.183 |
| 5 | Interior Plains | Interior Low Plateaus | Highland Rim | GBR-100_0.05_0.8_2 | 0.642 |
|  |  | Great Plains | Central Texas | GBR-100_0.05_0.6_2 | 0.617 |
|  |  | Interior Low Plateaus | Nashville Basin | LIN_REG-4 | 0.538 |
|  |  |  | Lexington Plain | UNCORRECTED | 0.496 |
|  |  | Central Lowland | Till Plains | LIN_REG-1 | 0.464 |
|  |  | Great Plains | Edwards Plateau | LIN_REG-1-FLAT0 | 0.464 |
|  |  |  | High Plains | LIN_REG-1 | 0.394 |
|  |  |  | Colorado Piedmont | LIN_REG-1 | 0.346 |
|  |  | Central Lowland | Osage Plains | UNCORRECTED | 0.310 |
|  |  |  | Dissected Till Plains | UNCORRECTED | 0.262 |
|  |  | Great Plains | Pecos Valley | LIN_REG-3 | 0.247 |
|  |  |  | Black Hills | LIN_REG-1 | 0.059 |
|  |  | Central Lowland | Wisconsin Driftless | RIDGE-1 | 0.046 |
|  |  |  | Eastern Lake | LIN_REG-2 | 0.029 |
|  |  | Great Plains | Missouri Plateau, Glaciated | LIN_REG-2 | 0.010 |
|  |  | Central Lowland | Western Lake | GBR-200_0.05_0.6_2 | 0.009 |
|  |  | Great Plains | Plains Border | GBR-200_0.1_0.6_8-FLAT0 | -0.906 |
|  |  |  | Missouri Plateau, Unglaciated | LASSO-5 | -3.502 |
|  |  |  | Raton | LIN_REG-1-FLAT0 | -5.372 |
| 6 | Atlantic Plain | Coastal Plain | Mississippi Alluvial Plain | UNCORRECTED | 0.526 |
|  |  |  | Floridian | LIN_REG-3 | 0.423 |
|  |  |  | East Gulf Coastal Plain | LASSO-2-FLAT0 | 0.179 |
|  |  |  | Sea Island | LIN_REG-3-FLAT0 | -0.338 |
|  |  |  | Embayed | GBR-200_0.05_0.8_8-FLAT0 | -1.013 |
|  |  |  | West Gulf Coastal Plain | LASSO-1 | N/A |
| 7 | Pacific Mountain System | Lower Californian | Lower Californian | LIN_REG-1 | 0.437 |
|  |  | Pacific Border | Oregon Coast Range | GBR-100_0.05_0.8_2 | 0.408 |
|  |  | Cascade-Sierra Mountains | Southern Cascade Mountains | GBR-100_0.05_0.6_2 | 0.317 |
|  |  |  | Middle Cascade Mountains | LIN_REG-1 | 0.228 |
|  |  | Pacific Border | Olympic Mountains | GBR-100_0.05_0.8_2 | 0.108 |
|  |  |  | Klamath Mountains | UNCORRECTED | 0.106 |
|  |  |  | Puget Trough | LIN_REG-3-FLAT0 | 0.069 |
|  |  |  | California Coast Ranges | ELASTIC_NET-3-FLAT0 | 0.050 |
|  |  | Cascade-Sierra Mountains | Northern Cascade Mountains | GBR-100_0.05_0.8_2 | -0.017 |
|  |  |  | Sierra Nevada | LASSO-3-FLAT0 | -0.050 |
|  |  | Pacific Border | California Trough | UNCORRECTED | -1.600 |
|  |  |  | Los Angeles Ranges | LASSO-1 | N/A |

­­­*For linear models the number following the estimator’s name is the maximum polynomial degree used for transforming uncorrected runoff for training and prediction.

** For *GBR* the trailing numbers indicate the following hyper parameter values: number of estimators in the ensemble, the boosting learning rate, the fraction of the training sample to use for stochastic gradient descent, and the maximum tree depth.

*** The suffix *FLAT0* indicates that a model predicts zero runoff for uncorrected zero runoff days instead of using the mean of observed runoff from those days.
